# Some Surgical Work in San Antonio

**Published:** 1894-09

**Authors:** R. Menger

**Affiliations:** City Physician; San Antonio, Texas


### Some Surgical Work in San Antonio.

LETTER FROM CITY PHYSICIAN R. MENGER.

SAN ANTONIO, TEXAS, Sept. 1, 1894.

*Editor Texas Medical Journal:*

At your request I will drop you a few lines concerning the few interesting surgical cases that occurred here, and related to you, lately in the hospitals and outside of same. Dr. Hadra operated, about two months ago, on an old citizen for gallstones, removing several large calculi, the man recovering completely (after being treated for years for different diseases, and being sent to Carlsbad, Germany, etc., without the least benefit).

The most interesting operative case in abdominal surgery occurred three weeks ago, when Drs. Braunnagel and A. Herff resected the lower colon for intussusception and cancerous degeneration, removing fully six inches of the bowel and stitching both ends together. The case was, in many respects, a rare and very interesting one. It concerned an Italian about fifty years old, who had been suffering for about one year with a peculiar bowel complaint on the left side of the abdomen. There was a nodulated tumor to be felt through the abdominals walls, but what it exactly was or could be, was, as generally in similar cases, only guesswork, but, at any rate, it could be traced to the lower colon, near the sigmoid flexure. Of late the man had much trouble in moving the bowels, the centents being somewhat bloody. On opening the abdomen at the site of the tumor, it was with much difficulty, on account of adhesions, etc., to get the same into view, and only after bringing a large portion of the gut outside the abdomen, it was noticed that the upper part of the bowel was incarcerated in the lower one, very much resembling a paraphymosis in its highest state of constriction. Considerable effort was made to reduce the constriction without resection, but, although quite a large piece of the upper bowel was replaced, it was utterly impossible to reduce it completely on account of old adhesions and (from all appearances) cancerous degeneration of the constricted bowel. A complete resection, therefore, of the constricted colon was made after tying both ends close to the tumor with a broad band. The stitching of both ends together was a very tedious piece of surgery, as the upper bowels were full with fæcal matter, and every now and then the patient, although under narcosis, would, from pressure of the diaphragm, etc., evacuate large quautities of bowel contents over the operating field. Both resected bowel-ends, however, were stitched complete before the abdomen was closed. The patient rallied well, although several whisky-hypodermics had to be given during the operation. On the second and third day there was no rising of temperature, and he had moved his bowels normally (the first time since nearly a year)! He progressed favorably and, with the exception of a very small amount of fæcal matter, which leaked through some of the stitch spaces, the man is rapidly recovering (now the third week).

Dr. A. Herff tells me that he had performed his nineteenth ovariotomy yesterday, with an uninterrupted record of recovery. This does not include a large number of other abdominal cases in which he also has had uniform success.

The better results in abdominal surgery of late years undoubtedly are much due to the better hospital equipments and trained nurses, and especially to the aseptic precautions observed in every case. We have nearly every day several large surgical cases on hand. Three days ago Dr. Withers removed a neuroma of the ischiatic nerve, the size of a large fist, in the popliteal region of the left leg, in a negro—*without chloroform!*—at the county hospital. The negro would rather die on the table than take chloroform, so we relieved him, although under excruciating pain, without it. The tumor was situated in and a little above the popliteal space (straight in the middle line), and it was quite a tedious job to extirpate the same. It was of a light bluish color and medullary appearance on dissection of same, after its extirpation. We thought, of course, also more of an aneurismatic tumor at the time of operation, but cut following both ends of the tnmor, unmistakably as the ischiatic nerve. The interesting feature in this case is that the negro, who is rapidly recovering, has lost none of the sensory or motor power, being able to move and lift his leg.

I made, yesterday morning, two abdominal operations, one for irreducible hernia, complicated with hydrocele, and one for an enormous dilated bladder, complicated with prostatitis, cystitis, etc., in a man about sixty years of age. On account of the prostatic complication and general polyuria besides, the urine had to be tapped very often during each day, and yesterday the bladder suddenly filled so enormously that it was impossible to pass a catheter. I relieved the condition by suprapubic cystotomy and drainage, similar to the case reported to your JOURNAL some years ago, in a young man with stab-wound in the bladder; the man recovering rapidly. But in this case, the one operated yesterday, I first stitched the wall of the bladder to the abdominal parieties, then opened the bladder and introduced a large rubber catheter, through the abdominal opening, into the bladder for irrigation and drainage purposes. The case is doing well so far.

